# Beyond HIV Shame: Effects of Self-Forgiveness in Improving Mental Health in HIV-Positive Individuals in Poland

**DOI:** 10.1007/s10943-024-02084-7

**Published:** 2024-08-03

**Authors:** Sebastian Binyamin Skalski-Bednarz, Loren L. Toussaint, Janusz Surzykiewicz

**Affiliations:** 1 Institute of Psychology, Humanitas University, Kilinskiego 43, 41-200 Sosnowiec, Poland; 2 Faculty of Philosophy and Education, Katholische Universität Eichstätt-Ingolstadt, Eichstätt, Germany; 3 Department of Psychology, Luther College, Decorah, IA USA; 4 Faculty of Education, Cardinal Stefan Wyszyński University in Warsaw, Warsaw, Poland

**Keywords:** Self-forgiveness, HIV, Heart rate, Quality of life, Health outcomes

## Abstract

Guided by the bio-psycho-socio-spiritual approach, this randomized controlled trial assessed the efficacy of a self-forgiveness intervention among 60 HIV-positive individuals in Poland. Participants underwent a 90-min "Restore: The Journey Toward Self-Forgiveness" session, in contrast to a wait-list control group. The intervention significantly enhanced self-forgiveness, spirituality, mental well-being, and heart rate variability in response to a cognitive stressor (i.e., a mental arithmetic challenge). Significant effects were observed in both between-group and within-subject comparisons. These results support the incorporation of self-forgiveness into psychological rehabilitation programs for HIV to improve quality of life and health outcomes.

## Introduction

The global impact of HIV continues to be profound, affecting millions worldwide (Dobrakowski & Skalski, 2019). Despite advancements in medical treatment transforming HIV into a manageable chronic condition (Bloch et al., 2020), the diagnosis still carries significant psychological burdens including stress, depression, death anxiety, reduced life satisfaction, and mood disorders (Ayano et al., 2020; Castilho et al., 2020; Chukwuorji et al., 2020; Rzeszutek & Gruszczyńska, 2023). These strains are often exacerbated by self-condemnation, particularly among individuals who perceive their infection as a result of socially or morally questionable behaviors (Bayat et al., 2020; Lyons et al., 2023; Toussaint et al., 2023).

Religious and spiritual beliefs have the potential to influence how individuals deal with guilt and moral failure within the bio-psycho-socio-spiritual approach, a holistic model integrating these factors to address overall health and well-being (Wang, 2011). Religious teachings on forgiveness and grace can provide comfort and help alleviate self-condemnation (Exline et al., 2017). Furthermore, support from religious communities aids in managing these complex emotions, offering social support for repentance and moral repair, and promoting a balanced view of personal failings (Wilt et al., 2016). This perspective not only addresses the physical and psychological aspects of living with HIV but also fosters spiritual growth and emotional healing.

### Self-Forgiveness as a Coping Mechanism

Self-condemnation in HIV-positive individuals often arises from internalized stigma and perceived personal responsibility for contracting the infection (Rueda et al., 2016). This self-directed negativity can intensify stress, leading to severe mental health challenges such as depression, anxiety, and decreased life satisfaction (Martin et al., 2012). Self-forgiveness is a crucial mechanism for mitigating these effects, offering a pathway out of the cycle of self-condemnation and facilitating improved psychological adjustment (Eaton et al., 2020; Peterson et al., 2017; Strelan et al., 2017; Toussaint et al., 2014, 2017; Webb et al., 2017; Webb & Toussaint, 2018). It involves releasing oneself from negative emotions and self-blame associated with past transgressions or mistakes, ultimately restoring self-worth and inner peace (Wohl et al., 2008).

Religious beliefs often motivate the process of self-forgiveness, emphasizing forgiveness, grace, and redemption. Many religious traditions encourage individuals to seek forgiveness not only from others and a higher power but also from themselves, viewing self-forgiveness as an essential step toward spiritual growth and reconciliation with one's faith (Escher, 2013; Fincham et al., 2020). In this context, religious teachings can provide a powerful framework for individuals to understand and practice self-forgiveness, thereby enhancing their psychological and spiritual well-being.

The *stress-and-coping model of self-forgiveness*, formulated by Toussaint et al. (2017), suggests that self-forgiveness acts as an emotion-focused coping mechanism to mitigate stresses related to personal failures, guilt, or discrepancies between one's values and behaviors. This model extends the transactional theory of stress and coping (Lazarus & Folkman, 1984) by incorporating self-forgiveness as a strategy to reduce the stress of self-condemnation, thereby enhancing overall health through the mediation and moderation of negative self-directed emotions. For HIV-positive individuals, self-forgiveness can lessen self-stigma, reduce feelings of shame and isolation (Zulkarnain et al., 2020), and foster acceptance of their health condition. This acceptance promotes adherence to healthcare routines, including antiretroviral therapy (ART) and other prescribed treatments, resulting in improved health management (Foster et al., 2020; Horter et al., 2017). Additionally, self-forgiveness is linked to enhanced mental health outcomes, notably reducing symptoms of depression and anxiety prevalent among those living with HIV (Ayano et al., 2020, 2021; Martin et al., 2012).

### Toward the Power of Self-Forgiveness

Empirical studies have shown that self-forgiveness facilitates acceptance of HIV status, enhances adherence to medical care, and supports openness in disclosing the condition (Nkomo & Kufankomwe, 2020; Skalski-Bednarz et al., 2024; Toussaint et al., 2023). Research also underscores the relationship between spirituality and self-forgiveness, fostering a holistic healing environment that enhances mental and spiritual well-being (Skalski-Bednarz et al., 2024). Mental well-being refers to a state of psychological health where an individual can cope with stress, work productively, and contribute to their community (Topp et al., 2015), while spiritual well-being involves a sense of peace and fulfillment derived from one's connection to a higher purpose or values (Surzykiewicz et al., 2022).

The "Restore: The Journey Toward Self-Forgiveness" program, a 90-min educational session, promotes self-reflection and therapeutic activities and has proven effective in addressing self-condemnation through self-forgiveness in clinical trials (Toussaint et al., 2014). This intervention could benefit HIV-positive individuals if appropriately adapted and tested. Besides its utility in various clinical groups, verifying the effectiveness of "Restore" in underrepresented non-American populations is crucial to demonstrate the cross-cultural validity of self-forgiveness. Poland, a culturally Catholic country, is particularly interesting because spiritual care is an important part of general health care, emphasizing integrated healing approaches (Pawlikowski et al., 2022). Spirituality, as defined by Surzykiewicz et al. (2022), involves a connection to something greater than oneself, fostering resilience and psychological well-being. Self-forgiveness programs like "Restore" highlight the importance of spiritual and psychosocial factors in comprehensive health management (Cornish & Wade, 2015; Griffin et al., 2015; Peterson et al., 2017; Toussaint et al., 2024).

### Cardiovascular Health

Self-forgiveness interventions may benefit cardiovascular health, in addition to psychological well-being. Research links self-forgiveness and forgiveness of others to improved physiological outcomes, such as reactivity, recovery, and myocardial perfusion, suggesting cardiovascular benefits (Friedberg et al., 2007; Lawler-Row et al., 2008; Whited et al., 2010). Experimental evidence is limited, but Waltman et al. (2009) found that forgiveness therapies reduced myocardial perfusion defects from anger-recall stress, improving heart rate variability (HRV) and cardiovascular health.

HRV measures the variation between heartbeats and reflects autonomic nervous system balance, inversely related to stress, anxiety, and depression, indicating stress regulation capacity (Cheng et al., 2022; da Estrela et al., 2021). Psychological interventions improving self-regulation positively affect HRV and cardiovascular stress responses, highlighting progress in stress management (Murdock et al., 2017; Whited et al., 2014). HRV offers a reliable index for the biopsychological changes from self-forgiveness interventions, necessitating further research.

### Current Study

This paper uses Toussaint et al.'s (2017) stress-and-coping model of self-forgiveness to explore self-forgiveness as a coping strategy for emotional distress, particularly in the context of HIV, which involves substantial psychological stress from stigma and chronic condition management (Ayano et al., 2020; Rueda et al., 2016). Self-forgiveness reduces negative emotions, fosters acceptance, and improves coping, potentially enhancing health-related quality of life (Peterson et al., 2017; Strelan et al., 2017; Webb et al., 2017).

Self-forgiveness interventions can help individuals with chronic conditions like HIV by fostering acceptance, improving mental well-being, spiritual growth, life satisfaction, and cardiovascular health (measured by HRV), and reducing depressive symptoms and death anxiety. Our study assessed the immediate effects of the "Restore" intervention (Toussaint et al., 2014) on HIV-positive individuals in Poland, hypothesizing significant health improvements post-intervention. We also hypothesized that religious patients would experience greater effects due to the supportive role of their beliefs and communities in promoting forgiveness and reducing self-condemnation (Exline et al., 2017). This research advances the understanding of HIV management's psychosocial aspects and supports developing comprehensive interventions for chronic conditions.

## Materials and Methods

### Participants

This randomized controlled study, conducted in the summer of 2023, received approval from the Scientific Research Ethics Committee of the University of Economics and Human Sciences in Warsaw (Approval No. 2/06/2023) and adhered to the World Medical Association Declaration of Helsinki guidelines. All participants provided written informed consent. The participant group included 60 HIV-positive individuals, all of whom were White Caucasians, with ages ranging from 20 to 37 years (*M* = 27.5, *SD* = 4.7). The demographic composition of the sample is presented in Table 1. All the participants were engaged in ART and maintained stable health, evidenced by CD4 + levels above 350/μl and undetectable viral loads.

**Table 1:** Participant demographic characteristics

Variable	%
*Gender*	
Cisgender man	77
Cisgender woman	23
*Religion*	
Roman catholic	62
No religious affiliation	38
*Race*	
White-caucasian	100
*Marital Status*	
Married	12
Separated or divorced	6
Never married	82
*Education*	
Primary	3
Secondary	51
Tertiary	46

Recruitment was carried out at an HIV care provider clinic in Krakow, Poland. The sole inclusion criterion for the study was a confirmed diagnosis of HIV. Recruitment strategies included advertisements and internal promotions within the clinic. Informed consent was obtained from all the participants who were fully briefed about the study requirements, location, and schedule well in advance. Each participant received compensation of PLN 50 for their time and involvement. Subsequently, the participants were randomly assigned to either the intervention group, who experienced the "Restore" program (Toussaint et al., 2014), or a wait-list control group.

### Procedure

Participants in this randomized controlled trial were randomly assigned to either the intervention group or the wait-list control group. The randomization process was carried out using a computer-generated random number sequence to ensure unbiased assignment. Each participant was assigned a unique identifier, and these identifiers were input into a random number generator. The randomly generated numbers determined the group allocation. Participants who received random odd numbers were assigned to the intervention group and participants who received random even numbers were assigned to the control group.

The intervention group participated in a 90-min session called “Restore: The Journey Toward Self-Forgiveness” (Toussaint et al., 2014), led by a licensed psychologist. This program was designed to foster self-forgiveness by engaging participants in structured writing exercises that encouraged personal reflection. Although the activities were completed individually, the psychologist monitored group dynamics and promoted active participation. Concurrently, the control group was involved in passive activities, such as board games and reading, to maintain consistent environmental conditions. Both groups met in small sessions of three to five individuals at a psychology center located near the HIV care clinic. The data were collected over 3 days.

**Fig. 1:**
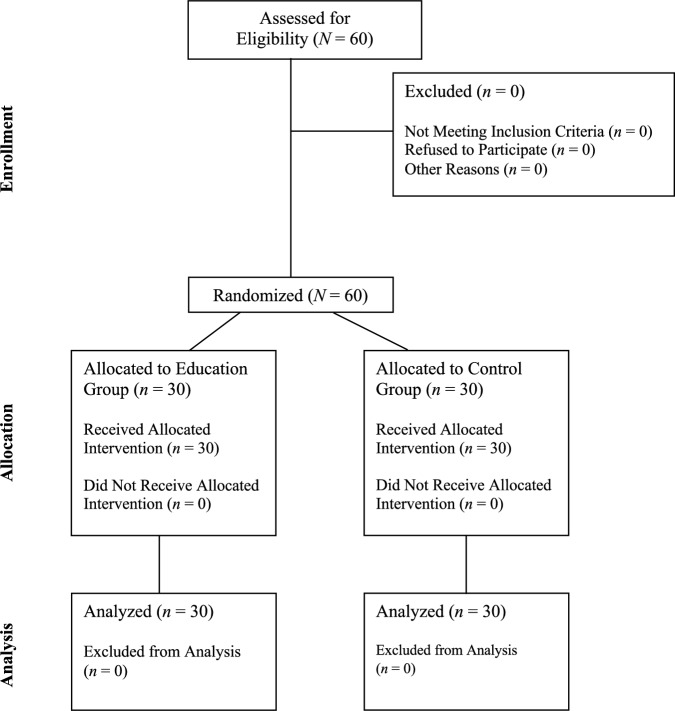
CONSORT flow diagram for a randomized controlled trial

Both groups of participants completed anonymous questionnaires using paper and pencil during their regular consultations 3 days prior to (i.e., pre) and immediately following (post) the intervention. These questionnaires assessed various psychological factors, including self-forgiveness, spirituality, acceptance of HIV status, mental well-being, life satisfaction, depressive symptoms, and death anxiety. After survey completion, the participants underwent HRV monitoring while performing a mental arithmetic challenge. Positioned in a comfortable armchair and facing a 19-in. screen approximately 70 cm away, the participants calculated the sum of two randomly selected integers between 1 and 49 under a 7 s time constraint. The result required a single keystroke input, achieved by adding the digits in the 1 s and 10 s places of their total. This task spanned 5 min, and each assessment session lasted about 30 min in total. The participant flow through the study phases is illustrated in the CONSORT flow diagram (see Fig. 1).

**Fig. 2:**
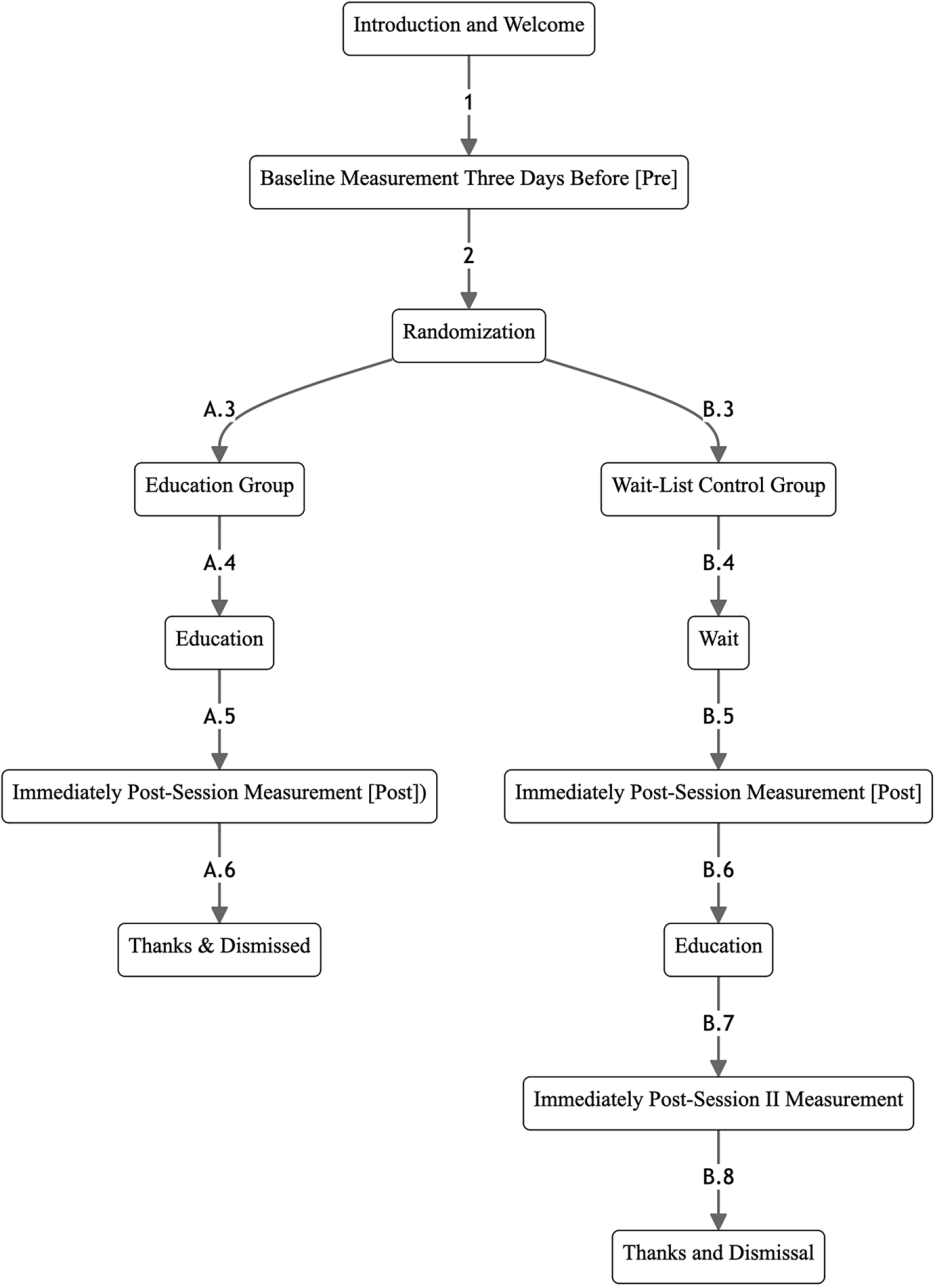
Study timeline

After the study, the wait-list participants were offered the self-forgiveness education and engaged in an additional session, followed by another set of questionnaires including extended HRV measures. The timeline for these activities is detailed in Fig. 2.

### Self-Forgiveness Education

The "Restore" program (Toussaint et al., 2014) is designed to facilitate self-forgiveness and personal growth through a structured educational curriculum (full access to a workbook available at discoverforgiveness.org). This program incorporates a workbook featuring various exercises and writing tasks that enable participants to engage deeply with concepts of self-unforgiveness and self-forgiveness. Self-unforgiveness is characterized by negative self-directed emotions such as hatred, anger, and regret, while self-forgiveness involves transforming these emotions to foster a positive self-image.

Participants start by acknowledging past actions that have led to feelings of guilt or remorse. This acknowledgment is followed by reflective exercises aimed at promoting acceptance and catalyzing personal change. The program also includes practices such as prayer, meditation, and quiet reflection, which are intended to deepen participants' engagement with the material and alleviate negative self-directed emotions. These activities collectively aim to enhance psychological well-being and empower individuals to make a positive impact on their communities.

An integral part of the workbook, "Benefit Finding," encourages participants to recognize how challenges can positively affect their relationships, faith, and appreciation for life. This process promotes a renewed sense of purpose and a commitment to community contribution. The program culminates with participants pledging to apply the insights gained toward their personal transformation and to assist others by leveraging their personal experiences to support those facing similar challenges.

Initially developed in English (Toussaint et al., 2014), the workbook was adapted into Polish by a proficient translator and validated by four Polish-speaking psychologists with expertise in forgiveness research. This adaptation process ensured the program's accessibility and cultural relevance to Polish-speaking participants.

Additional pages in the workbook allow for extensive reflection, enabling participants to progress at their own pace and fully immerse themselves in the transformative process of self-forgiveness. This comprehensive approach underscores the significance of self-forgiveness in overcoming personal hurdles and enhancing well-being across physical, emotional, and spiritual dimensions.

### Measures

#### State Self-Forgiveness Scale (SSFS)

Developed by Wohl et al. (2008) and adapted into Polish by Mróz and Sornat (2021), the SSFS is a 17-item instrument designed to assess state self-forgiveness. Recognized as one of the few validated tools for measuring immediate self-forgiveness experiences, the scale evaluates two dimensions: self-forgiving feelings and actions, and self-forgiving beliefs. In its Polish version, responses are captured on a four-point Likert scale from 1 (not at all) to 4 (completely). Sample items include "I forgive myself for my mistakes" and "I believe I am worthy of love despite my flaws." The SSFS demonstrated high internal consistency in this study (*α* = 0.89, reported at the pre-intervention measurement point). For this variable, and for all subsequent variables mentioned in this manuscript, a higher score signified increased levels of the construct. Throughout this document, we consistently report average values. The scale's strong factorial and predictive validity was confirmed by studies showing positive correlations with mental health indicators (Mróz & Sornat, 2021; Wohl et al., 2008).

#### Interfaith Spirituality (IFS) Scale

Originally developed by Kira et al. (2021) and later standardized for Polish populations by Surzykiewicz et al. (2022), the IFS scale measures spirituality within an interfaith context. It assesses spirituality through indicators of a direct connection to a divine presence and the ability for self-transcendence. Participants rated four statements on a four-point scale from 1 (*definitely not*) to 4 (*definitely yes*), with higher scores reflecting a stronger spiritual connection. Sample items include "I feel a deep connection with my Creator" and "I often think about the broader meaning of life" (all items are presented in "Appendix 1"). The reliability of the IFS scale in this study was confirmed with a Cronbach’s alpha of 0.94 (pre-intervention measurement). Additionally, Surzykiewicz et al. (2022) observed that the interfaith spirituality approach can be effectively applied to both believer and non-believer populations in Poland.

#### Acceptance of Illness Scale (AIS)

Developed by Felton et al. (1984) and translated into Polish by Czerw et al. (2022) and specifically adapted for an HIV context, the AIS measures acceptance of medical conditions. It includes eight items that explore the impact of the infection, such as health limitations and dependency on others. Responses are scored on a five-point Likert-type scale from 1 (*completely agree*) to 5 (*completely disagree*). Sample items include “My HIV-positive status does not define me” and “I am able to accept the limitations imposed by my HIV-positive status.” The AIS is noted for its robust reliability, evidenced by a Cronbach’s alpha of 0.91 at the pre-intervention measurement point.

#### World Health Organization Well-Being Index (WHO-5)

Originally developed by Topp et al. (2015) and adapted into Polish by Cichoń et al. (2020), this five-item screening tool assesses psychological well-being, with higher scores indicating better psychological health. Participants respond on a six-point Likert scale reflecting their experiences over the past 2 weeks, from 0 (*at no time*) to 5 (*all the time*). Sample items include “I have felt cheerful and in good spirits” and “I felt calm and relaxed.” The scale’s reliability and validity are supported by a Cronbach’s alpha of 0.89 at the pre-intervention measurement point and by positive correlations with health and life satisfaction, as shown in the validation study by Cichoń et al. (2020).

#### Satisfaction with Life Scale (SWLS)

Designed by Diener et al. (1985) to measure life satisfaction, the SWLS includes five statements rated on a seven-point Likert scale from 1 (*I strongly disagree*) to 7 (*I strongly agree*). Sample items include “In most ways, my life is close to my ideal” and “The conditions of my life are excellent.” The Polish adaptation by Juczyński (2009) demonstrated satisfactory reliability with a Cronbach’s alpha of 0.86 at the pre-intervention measurement point.

#### Patient Health Questionnaire (PHQ-9)

Developed by Kroenke et al. (2001) to evaluate depressive symptoms, the PHQ-9 was adapted for Polish populations by Kokoszka et al. (2016). It includes nine items based on DSM-5 criteria for depression, with responses rated from 0 (*not at all*) to 3 (*almost every day*). Sample items include “Feeling down, depressed, or hopeless?” and “Trouble falling or staying asleep, or sleeping too much?” The scale’s unidimensional reliability was confirmed with a Coefficient alpha of 0.88) at the pre-intervention measurement point.

#### Death Attitude Profile-Revised (DAP-R), Fear of Death Subscale

Developed by Wong et al. (1994) and adapted into Polish by Brudek et al. (2020), this subscale assesses an individual’s fear of death via seven items on a seven-point scale from 1 (*strongly disagree*) to 7 (*strongly agree*). The reliability Coefficient alpha of 0.83 at the pre-intervention measurement point was acceptable. The scale explores perceptions of death as an unknown and anxiety-provoking experience. Sample items include “I am afraid of dying” and “Thinking about death fills me with dread.”

#### Heart Rate Variability (HRV)

Following the completion of the questionnaires, HRV data were collected using a Vernier LabQuest® Mini (Model 2) and recorded through Logger Pro™ (Version 3.16.2). Electrocardiography electrodes were strategically placed on the participants’ arms—one on the right wrist, another at the right elbow, and a third at the left elbow. These placements were maintained throughout the arithmetic challenge to ensure continuous recording of heart rate data during this intellectual stressor.

The HRV analysis was conducted using Kubios HRV Scientific (Version 4.0.3), a tool developed by the Biosignal Analysis and Medical Imaging Group at the University of Eastern Finland in Kuopio, Finland. The standard deviation of normal-to-normal (SDNN) intervals was utilized as the primary HRV measure, in line with recommendations by the Task Force of the European Society of Cardiology and the North American Society of Pacing Electrophysiology (1996). Despite the prevalent use of SDNN intervals, the interpretation of frequency domain variables remains contentious due to the lack of consensus in the field regarding their implications on sympatho-vagal balance (Billman, 2013).

A systematic review by Nunan et al. (2010) indicated that the average SDNN intervals across 27 studies was 50 ms, with a median of 51 ms and a range of 32–93 ms. Medicore’s Heart Rate Variability Analysis System categorizes an average SDNN above 50 ms as "high normal," suggesting effective autonomic nervous system regulation and resilience against stress-related conditions. Conversely, values below 35 ms are considered indicative of poor regulatory function and an elevated risk for stress-induced illnesses (Medicore, 2024).

Accurate HRV assessment is susceptible to errors from technical and physiological artifacts (Peltola, 2012). Technical issues may include incorrect QRS detection or imprecise RR interval measurements due to software errors or improper electrode placement. Physiological issues might encompass ectopic beats among other arrhythmias. To counter these artifacts, Kubios offers robust correction techniques, including piecewise cubic spline interpolation, which was utilized in this study with a threshold of 0.05 s for abnormal RR interval deviations (Tarvainen & Niskanen, 2012).

Despite advanced artifact correction, participant movement during this study’s arithmetic challenge led to significant technical artifacts. Additional physiologically improbable data points were likely due to electrode dislodgement. These outliers were removed, and the remaining data were log-transformed to normalize the distribution, ensuring the validity of our measurements.

### Statistical Analysis

The distributions’ normality was confirmed using the Kolmogorov–Smirnov test, and variance equality was checked with Levene's test, enabling the use of parametric tests. Differences were evaluated using a multivariate repeated measures ANOVA, with simple main effects and contrasts analysis. Mauchly's test assessed sphericity. Relationships among variables were explored through the Pearson correlation matrix. Effect sizes were determined using partial *η*2 and Cohen's *d*. The minimum sample size required for the study was calculated using G*Power (Version 3.1.4). All data analyses were performed using IBM’s SPSS (Version 29).

## Results

The mean values of the variables in this study were assessed among HIV-positive individuals at two measurement points: pre and post. Table 2 presents the pairwise comparisons of the estimated marginal means, adjusted for multiple comparisons using the Bonferroni method. A 2 × 2 repeated measures ANOVA evaluated the effects of the "Restore" program. The between-subjects factor, *education*, compared participation in the "Restore" training with a non-intervention wait-list control group. The within-subjects factor, *time*, included pre- and post-measurement points. The dependent variables were self-forgiveness, spirituality, HIV status acceptance, mental well-being, life satisfaction, depressiveness, death anxiety, and HRV, measured by SDNN intervals. Additionally, gender and age were not significantly related to the results, *p* > 0.05.

**Table 2:** Mean scores by time and group

Outcome	Group	Pre *M* (*SD*)	Post *M* (*SD*)	Within-subject comparison	Between-group comparison
Self-forgiveness	Intervention	1.9 (1.0)	2.5 (1.3)	Pre < post***	Pre: ns
	Control	2.0 (0.9)	1.9 (0.6)	ns	Post: education > control*
Spirituality	Intervention	1.9 (0.9)	2.3 (0.8)	Pre < post*	Pre: ns
	Control	1.8 (0.5)	1.8 (0.7)	ns	Post: education > control**
HIV status acceptance	Intervention	2.7 (1.5)	3.4 (1.3)	Pre < post**	Pre: ns
	Control	2.8 (1.5)	2.7 (1.4)	ns	Post: education > control*
Mental Well-being	Intervention	3.1 (1.8)	4.1 (1.7)	Pre < post**	Pre: ns
	Control	3.3 (1.9)	3.1 (1.6)	ns	Post: education > control*
Life satisfaction	Intervention	2.5 (1.4)	3.1 (1.4)	Pre < post***	Pre: ns
	Control	2.6 (1.3)	2.4 (1.4)	ns	Post: education > control*
Depressiveness	Intervention	1.6 (1.1)	1.1 (1.0)	Pre > post***	Pre: ns
	Control	1.7 (1.1)	1.6 (1.0)	ns	Post: education < control*
Death anxiety	Intervention	3.9 (0.9)	3.6 (0.8)	Pre > post*	Pre: ns
	Control	3.9 (1.0)	3.9 (0.9)	ns	Post: education < control*
HRV (SDNN)	Intervention	42.9 (13.3)	47.6 (13.1)	Pre < post*	Pre: ns
	Control	41.7 (13.9)	43.1 (14.1)	ns	Post: education > control*

*HRV* heart rate variability, *SDNN* standard deviation of the normal-to-normal intervals, *SD* standard deviation. Statistical significance: *p ≤ 0.05; **p ≤ 0.01; ***p ≤ 0.001; ns indicates not significant

Our analyses revealed statistically significant multivariate effects: a main effect for *education*, *F*_(8, 51)_ = 3.96, *p* = 0.001, *η*^2^ = 0.38, and an interaction effect for *education* and *time*, *F*_(8, 51)_ = 4.36, *p* = 0.001, η^2^ = 0.41. The main effect for *time* was not significant, *p* > 0.05. Given the significant multivariate interaction, univariate tests were conducted for the interaction between *education* and *time* across all dependent variables. Univariate tests confirmed the interaction effects for self-forgiveness, *F*_(1, 58)_ = 9.14, *p* = 0.004, *η*^2^ = 0.14; spirituality, *F*_(1, 58)_ = 4.21, *p* = 0.045, *η*^2^ = 0.07; HIV status acceptance, *F*_(1, 58)_ = 5.67, *p* = 0.021, *η*^2^ = 0.09; mental well-being, *F*_(1, 58)_ = 6.62, *p* = 0.013, *η*^2^ = 0.10; life satisfaction, *F*_(1, 58)_ = 4.37, *p* = 0.041, *η*^2^ = 0.07; depressiveness, *F*_(1, 58)_ = 6.14, *p* = 0.016, *η*^2^ = 0.10; death anxiety, *F*_(1, 58)_ = 4.16, *p* = 0.046, *η*^2^ = 0.07; and HRV, *F*_(1, 58)_ = 4.02, *p* = 0.05, *η*^2^ = 0.06. Our analyses examining whether the self-forgiveness intervention had a stronger effect for religious patients compared to nonreligious patients revealed no statistically significant multivariate main or two-way or three-way interaction effects (*p*s > 0.05).

### Analysis of Simple Main Effects

#### Between-Group Comparison of Intervention Effects

To clarify the observed interaction effects, an analysis of simple main effects was performed. This revealed that self-forgiveness education significantly and beneficially impacted all dependent variables at post-measurement, as compared to the wait-list group. The results showed significant improvements in self-forgiveness, *p* = 0.017, *d* = 0.59; spirituality, *p* = 0.007, *d* = 0.66; HIV status acceptance, *p* = 0.022, *d* = 0.53; mental well-being, *p* = 0.031, *d* = 0.49; life satisfaction, *p* = 0.039, *d* = 0.47; depressiveness, *p* = 0.021, *d* =  − 0.54; death anxiety, *p* = 0.048, *d* =  − 0.42; and HRV, *p* = 0.049, *d* = 0.33. No differences between the groups were evident at pre measurement (*p*s > 0.05, *d*s < 0.19).

#### Within-Subject Comparison of Intervention Effects

**Fig. 3:**
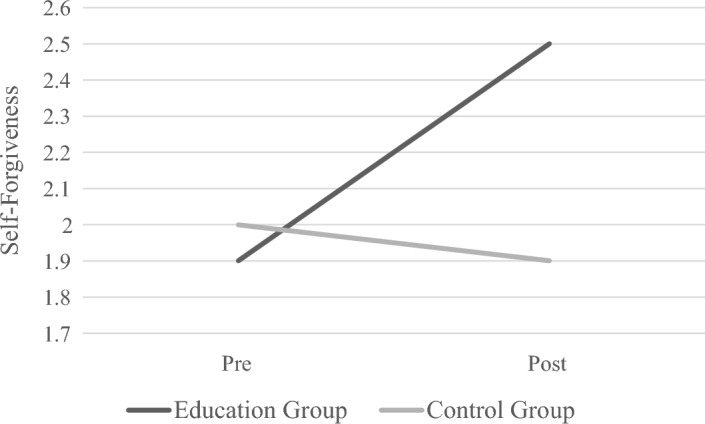
Differences in self-forgiveness between education and control groups

Beneficial changes in dependent variables for the "Restore" education program were statistically significant, including self-forgiveness, *p* < 0.001, *d* =  − 0.97; spirituality, *p* = 0.017, *d* =  − 0.38; HIV status acceptance, *p* = 0.006, *d* =  − 0.41; mental well-being, *p* = 0.004, *d* =  − 0.41; life satisfaction, *p* < 0.001, *d* =  − 0.72; depressiveness, *p* < 0.001, *d* = 0.55; death anxiety, *p* = 0.012, *d* = 0.64; and HRV, *p* = 0.037, *d* =  − 0.48. No changes were observed in the wait-list group *p*s > 0.05, *d*s < 0.12). Figure 3 illustrates the interaction of group (education vs. wait-list) and time (pre- vs. post-education) by providing the graphical results of the self-forgiveness outcome, as an example.

### Wait-List to Education: Within-Subject Comparisons

This study included a wait-list control group that was subsequently given the opportunity to engage in the "Restore" program after meeting earlier control conditions. Initially, 30 participants were randomized into the wait-list group, and 25 progressed to the educational phase upon fulfilling the control requirements. The analysis utilized data from pre-education and post-education conditions for the wait-list-turned-education group. The wait-list-turned-education group had originally not been subjected to the intervention or the follow-up HRV challenge and served merely as a comparison group to assess the impact of the "Restore" program. Continuous data collection from the original wait-list group offered a unique opportunity to evaluate the effectiveness of the self-forgiveness education by examining changes in scores. In the absence of a randomized comparison group at this stage, a repeated measures ANOVA without a between-subjects factor was employed to analyze the differences, focusing solely on the changes across time. Mauchly's test confirmed that the assumption of sphericity was not violated before the analyses.

A significant multivariate effect of “Restore” education was observed, *F*_(16, 82)_ = 4.44, *p* < 0.001, *η*^2^ = 0.46. Notable statistical changes were recorded in self-forgiveness, *F*_(1, 48)_ = 4.84, *p* = 0.012, *η*^2^ = 0.17; spirituality,* F*_(1, 48)_ = 5.07, *p* = 0.010, *η*^2^ = 0.17; HIV status acceptance, *F*_(1, 48)_ = 7.09, *p* = 0.002, *η*^2^ = 0.23; mental well-being, *F*_(1, 48)_ = 8.09, *p* < 0.001, *η*^2^ = 0.25; life satisfaction, *F*_(1, 48)_ = 5.68,* p* = 0.006, *η*^2^ = 0.19; depressiveness, *F*_(1, 48)_ = 5.81, *p* = 0.005, *η*^2^ = 0.20; death anxiety, *F*_(1, 48)_ = 5.20, *p* = 0.009, *η*^2^ = 0.19; and HRV, *F*_(1, 48)_ = 5.72, *p* = 0.006, *η*^2^ = 0.19. Subsequent tests of within-subject contrasts revealed significant improvements in all variables at Time 3 (*T*3) compared to *T*1 and *T*2. Detailed statistics are reported in Table 3.

**Table 3:** Within-subject contrasts for the wait-list control group before and after participation in the education program

	A. Pre *M* (SD)	B. Post *M* (SD)	C. Follow-up post-restore program *M (SD)*	Contrasts
Self-forgiveness	2.0 (1.0)	1.9 (0.7)	2.6 (1.4)	A < C*; B < C**
Spirituality	1.8 (0.6)	1.7 (0.8)	2.4 (1.0)	A < C*; B < C*
HIV status acceptance	3.0 (1.6)	2.8 (1.5)	3.8 (1.1)	A < C*; B < C**
Mental well-being	2.7 (1.4)	2.5 (1.5)	3.2 (1.3)	A < C*; B < C**
Life satisfaction	3.3 (1.9)	3.5 (1.9)	4.5 (1.3)	A < C**; B < C*
Depressiveness	1.6 (1.2)	1.6 (1.1)	0.9 (0.8)	A > C*; B > C*
Death anxiety	3.7 (0.9)	3.6 (0.7)	3.2 (0.5)	A > C*; B > C*
HRV (SDNN)	42.5 (14.2)	45.2 (13.5)	53.2 (15.9)	A < C**; B < C*

*HRV* heart rate variability, *SDNN* standard deviation of the normal-to-normal intervals, *SD* standard deviation. Statistical significance: *p ≤ 0.05; **p ≤ 0.01

## Discussion

This study explored the effects of the "Restore: The Journey Toward Self-Forgiveness" program developed by Toussaint et al. (2014) on various psychosocial and physiological metrics in HIV-positive individuals. Based on the stress-and-coping model of self-forgiveness (Toussaint et al., 2017), the “Restore” program leverages self-acceptance, self-improvement, and commitment as foundational pillars of self-forgiveness (Toussaint et al., 2014). In the present study, significant improvements were noted across several dimensions, including self-forgiveness, spirituality, acceptance of HIV status, mental well-being, life satisfaction, depressive symptoms, death anxiety, and HRV, confirming that self-forgiveness interventions can substantially benefit both psychological and physiological health outcomes. Significant effects were observed in both the between-group comparison post-intervention (education group vs. wait-list control group) and the within-subject comparison of pre- and post-intervention measures in the "Restore" participants.

The improvements were directly attributed to the educational program, not merely to the passage of time, as evidenced by the lack of significant changes in the wait-listed control group. This conclusion is bolstered by further supportive data from the wait-listed group when they were offered the “Restore” education, substantiating the beneficial effects of the "Restore" intervention (Toussaint et al., 2014). However, study outcomes were measured only immediately after the intervention, with no follow-up assessments to track longer-term effects. However, Cornish and Wade (2015)have noted that self-forgiveness gains from individual counseling were maintained at a two-month follow-up.

### Comparative Studies and Intervention Fit

Some previous studies have linked self-forgiveness to improved mental health outcomes across various populations (Cornish & Wade, 2015; Griffin et al., 2015; Strelan et al., 2017; Toussaint et al., 2014). The current study extends these findings to HIV-positive individuals, highlighting the program’s significant impact on participants’ negative self-perceptions and levels of personal acceptance. The effects observed in this study were moderate to large, consistent with Toussaint et al. (2014), who applied the same intervention to cancer patients and their caregivers. In their study, the impacts were notably large for self-forgiving feelings and moderate for self-acceptance, self-improvement, and pessimism.

Furthermore, Griffin et al. (2015) analyzed data from college students and proposed that the treatment’s effectiveness depends on the alignment between individual characteristics and the intervention’s activities, establishing the optimal conditions for successful outcomes. They specifically highlighted that individuals who benefit the most from the self-forgiveness workbook typically begin with low levels of self-forgiveness, perceive their offenses as particularly severe, and engage more actively with the educational content.

### Reducing Cardiovascular Risks in HIV

The program's effects underscore potential physiological benefits such as improved autonomic balance, crucial for reducing cardiovascular risks—a significant concern for HIV-positive individuals. Despite effective viral suppression, these individuals face an elevated risk of cardiovascular diseases, including myocardial infarction, stroke, and heart failure (Freiberg et al., 2017; So-Armah et al., 2020). With ART extending life expectancy, the incidence of age-related cardiovascular conditions also rises. Therefore, interventions like "Restore" (Toussaint et al., 2014) that enhance both psychological and physiological health could notably decrease cardiovascular risks in this population.

Additionally, our results lend support to the hypothesis that improvements in HRV are associated with self-forgiveness, consistent with enhanced physiological outcomes such as reactivity, recovery, and myocardial perfusion that are associated with forgiving others. These improvements may offer worthwhile benefits to cardiovascular health. This is supported by findings from Waltman et al. (2009), who demonstrated that forgiveness therapies significantly mitigated myocardial perfusion defects triggered by anger-recall stress, thus enhancing HRV and overall cardiovascular health (Waltman et al., 2009; Whited et al., 2010).

### Spirituality and Health Care

Conducted in Poland, a nation profoundly influenced by Roman Catholicism, this study examined spirituality among both believers and non-believers. We did not observe the expected greater effects of the intervention in religious patients. This diverges somewhat from what Escher (2013) found that Americans identifying with a Christian denomination were more likely to report self-forgiveness for past wrongdoings. Furthermore, we discovered no significant correlation between religious faith and spirituality at any measurement point, prompting further reflection. Christian values such as charity, solidarity, and community involvement permeate Polish society and influence even those who are not actively religious. These observations suggest a universally shared spiritual framework that transcends formal religious practices.

Surzykiewicz et al. (2022) proposed that a sense of spirituality, understood as a profound connection with a creator or superior energy and the capacity for self-transcendence, is widespread across the general population of Poland, regardless of the presence or absence of religious beliefs. Furthermore, Rye (2007) noted that participants in forgiveness programs, irrespective of the program's religious context, access their spiritual resources, fostering personal growth and reconciliation. This highlights the critical role of addressing spiritual needs in health care to improve treatment outcomes and patient well-being. The "Restore" program (Toussaint et al., 2014), aligned with findings from Oji et al. (2017), shows promise as an intervention that can significantly enhance the mental health and potentially medication adherence of HIV-positive populations.

### Cross-Cultural Applicability

This study demonstrated the effectiveness of the "Restore" education (Toussaint et al., 2014) within a Polish population, indicating its potential applicability across various cultural contexts. To date, self-forgiveness interventions have been primarily evaluated within English-speaking populations. Our findings cohere with existing research on forgiveness for others, which might suggest that both forgiveness of others and self-forgiveness interventions can be universally effective (Kurniati et al., 2020; Lin et al., 2014; Toussaint et al., 2020).

By extending this research to include a non-English-speaking European country, this study not only supports the cross-cultural utility of the "Restore" program (Toussaint et al., 2014) but also positions it as possibly suitable for global adaptation, potentially increasing its relevance and impact in diverse cultural settings. Although encouraging, these results represent a preliminary step, and further research is required to provide conclusive evidence that supports the widespread application of such interventions.

### Limitations

Our research effectively demonstrated the potential benefits of the "Restore: The Journey Toward Self-Forgiveness" program (Toussaint et al., 2014) through consistent self-reported improvements in self-forgiveness and health outcomes. Nonetheless, the use of immediate pre- and post-assessment methods restricted our insight into the long-term effects of the intervention. The relatively small sample size of less than 100 participants further limited the robustness and statistical power of our findings, potentially affecting the reliability of the results. Nevertheless, we did observe consistently moderate to large effect sizes allowing for a smaller sample size to provide sufficient statistical power and detect the hypothesized intervention effects. Future work should examine longer-term maintenance of the benefits of this self-forgiveness education.

Additionally, the study predominantly involved a homogenous group—primarily Caucasian, Christian males—which may constrain the generalizability of our findings to broader populations. Future studies should aim to include more diverse participants to validate the intervention’s effectiveness across various groups. Moreover, the similarity in treatment and health outcomes among participants prevented us from exploring these variables in greater depth.

Finally, the use of recognized scales like the SSFS and WHO-5 enhanced the credibility of our data. However, the reliance on self-reported mental health measures introduces biases that can distort the accuracy of results. Participants' emotional states, social desirability, or misunderstanding of questions can skew responses. Therefore, we incorporated HRV as an objective measure, which strengthened our findings by providing a quantifiable indicator of autonomic nervous system function less prone to subjective bias. Future studies of self-forgiveness interventions should continue to examine potential confounding of self-forgiveness self-report measures and take steps to minimize confounding effects.

## Conclusion

The "Restore: The Journey Toward Self-Forgiveness" intervention (Toussaint et al., 2014) may provide a significant enhancement to the existing psycho-socio-spiritual approaches for managing chronic conditions like HIV infection. By bolstering acceptance, psychological well-being, spirituality, and cardiovascular health, the program not only aligns with existing health strategies but also pioneers new pathways for comprehensive chronic disease management. To reinforce our encouraging results, further research with diverse populations and settings is needed to strengthen the evidence base that underscores the critical role of self-forgiveness interventions in health care.

## Data Availability

The datasets generated during and/or analyzed during the current study are available from the corresponding author on reasonable request.

## Appendix 1

### Statements from the Interfaith Spirituality Scale Employed in This Study

1. My feeling of direct connection with my creator gives me a sense of inner peace (Direct Connection with the Creator).
2. I meditate about the miracle of creation and the meaning of existence (Meditation and Spiritual Knowledge).
3. I have undescribed love for my creator (Divine Love).
4. Modesty, being realistic, and knowing my real value and limits promote my spiritual self (Asceticism, Self-discipline, and Virtues).
