# Correction: Beyond HIV Shame: Effects of Self-Forgiveness in Improving Mental Health in HIV-Positive Individuals in Poland

**DOI:** 10.1007/s10943-024-02114-4

**Published:** 2024-09-18

**Authors:** Sebastian Binyamin Skalski-Bednarz, Loren L. Toussaint, Janusz Surzykiewicz

**Affiliations:** 1 Institute of Psychology, Humanitas University, Kilinskiego 43, 41-200 Sosnowiec, Poland; 2 Faculty of Philosophy and Education, Katholische Universität Eichstätt-Ingolstadt, Eichstätt, Germany; 3 Department of Psychology, Luther College, Decorah, IA USA; 4 Faculty of Education, Cardinal Stefan Wyszyński University in Warsaw, Warsaw, Poland

**Correction to: Journal of Religion and Health** 10.1007/s10943-024-02084-7

In this article due to a mistake during the production process, Fig. 1 was incorrectly rendered, resulting in two lines which were missing while processing this figure in the graphics application.

**Fig. 1:**
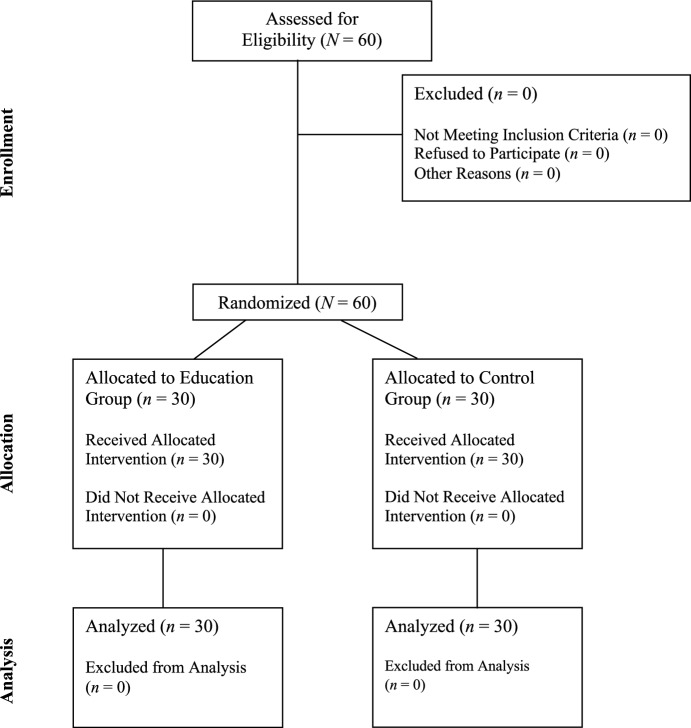
CONSORT flow diagram for a randomized controlled trial

The figure should have appeared as shown below.

The original article has been corrected.

